# Effect of Continuous and Discontinuous Microwave-Assisted Heating on Starch-Derived Dietary Fiber Production

**DOI:** 10.3390/molecules26185619

**Published:** 2021-09-16

**Authors:** Kamila Kapusniak, Karolina Lubas, Malwina Wojcik, Justyna Rosicka-Kaczmarek, Volodymyr Pavlyuk, Karolina Kluziak, Idalina Gonçalves, Joana Lopes, Manuel A. Coimbra, Janusz Kapusniak

**Affiliations:** 1 Department of Biochemistry, Biotechnology and Ecotoxicology, Faculty of Science and Technology, Jan Dlugosz University in Czestochowa, Armii Krajowej 13/15, 42-200 Czestochowa, Poland; k.kapusniak@ujd.edu.pl; 2 Department of Dietetics and Food Studies, Faculty of Science and Technology, Jan Dlugosz University in Czestochowa, Armii Krajowej 13/15, 42-200 Czestochowa, Poland; kwrobel292@gmail.com (K.L.); malwinawojcik96@gmail.com (M.W.); 3 Institute of Food Technology and Analysis, Faculty of Biotechnology and Food Sciences, Lodz University of Technology, Stefanowskiego 4/10 Street, 90-924 Lodz, Poland; justyna.rosicka-kaczmarek@p.lodz.pl; 4 Institute of Chemistry, Faculty of Science and Technology, Jan Dlugosz University in Czestochowa, Armii Krajowej 13/15, 42-200 Czestochowa, Poland; v.pavlyuk@ujd.edu.pl (V.P.); k.kluziak@ujd.edu.pl (K.K.); 5 CICECO—Aveiro Institute of Materials, Department of Materials and Ceramic Engineering, University of Aveiro, 3810-193 Aveiro, Portugal; idalina@ua.pt (I.G.); lopesjoana@ua.pt (J.L.); 6 LAQV-REQUIMTE, Department of Chemistry, University of Aveiro, 3810-193 Aveiro, Portugal; mac@ua.pt

**Keywords:** dietary fiber, resistant dextrin, potato starch, modified starch, microwave heating, molecular structure

## Abstract

Dietary fiber can be obtained by dextrinization, which occurs while heating starch in the presence of acids. During dextrinization, depolymerization, transglycosylation, and repolymerization occur, leading to structural changes responsible for increasing resistance to starch enzymatic digestion. The conventional dextrinization time can be decreased by using microwave-assisted heating. The main objective of this study was to obtain dietary fiber from acidified potato starch using continuous and discontinuous microwave-assisted heating and to investigate the structure and physicochemical properties of the resulting dextrins. Dextrins were characterized by water solubility, dextrose equivalent, and color parameters (L* a* b*). Total dietary fiber content was measured according to the AOAC 2009.01 method. Structural and morphological changes were determined by means of SEM, XRD, DSC, and GC-MS analyses. Microwave-assisted dextrinization of potato starch led to light yellow to brownish products with increased solubility in water and diminished crystallinity and gelatinization enthalpy. Dextrinization products contained glycosidic linkages and branched residues not present in native starch, indicative of its conversion into dietary fiber. Thus, microwave-assisted heating can induce structural changes in potato starch, originating products with a high level of dietary fiber content.

## 1. Introduction

Nowadays, there is growing interest in the correlation of nutrition with human health. Several studies have been conducted to develop functional foods that provide caloric intake and contain bioactive compounds [1]. Considering its beneficial effects on the human body, dietary fiber (DF) is considered to be part of a group of functional foods. Benefits of adequate intake of DF include prevention of diseases of civilization, such as obesity, cancer, cardiovascular diseases, and diabetes mellitus [2,3,4,5]. Some DF also promotes the growth of beneficial gut microbiota [6]. Despite the many advantages of DF consumption, its consumption is still below the recommended intake. The main sources of DF are vegetables, fruits, and whole grains. However, these products are still seldom consumed by populations of high-income countries. Improper habits resulting from the consumption of a Western-type diet are the cause of the aforementioned diseases of civilization [7]. For this reason, there is a need to develop new sources of DF that can be successfully added to food to obtain new health-promoting products of better quality and, above all, properties acceptable to consumers.

DF preparations were previously obtained by dextrinization, which occurs while heating starch in the presence of acids [8]. During dextrinization, depolymerization, transglycosylation, and repolymerization occur. As a result of these alterations, new linkages are formed. The decrease in α1,4- and α1,6-glucosidic digestible linkages, and the increase in non-digestible linkages 1,2- and 1,3-glycosidic in either α- or β-anomers have been observed [9,10,11,12]. This process allowed dextrins resistant to human digestive enzymes to be obtained [13]. Resistant dextrins have been classified as dietary fiber because of their beneficial effect on human health [14]. In recent studies, researchers have used conventional heating to produce starch-derived resistant dextrins. However, the processes were relatively time and energy consuming [13,15,16]. Using microwave irradiation as an energy source seems to offer great possibilities for overcoming these drawbacks.

Microwave irradiation is referred to as a type of electromagnetic irradiation in the frequency range from 0.3 to 300 GHz and wavelength from 1 mm to 1 m [17]. Microwave irradiation causes the movement of polar molecules or ions. The molecular friction induces energy loss from molecules in the form of heat. For this reason, microwave irradiation has been used for heating different materials [18]. Microwave heating has been reported to shorten the time of various reactions by 10 to 1000 times [19]. Moreover, it is known that microwave irradiation has high repeatability and high efficiency of uniform heating in the whole heated material.

Microwave irradiation has already been used in starch modification [20,21,22], in which it has allowed starch to be modified in a shorter time than conduction heating [23]. Microwave heating has also been proposed and it has been successfully used to obtain enzyme-resistant dextrins [24]. In the majority of conducted research, common domestic microwave ovens as radiation source were used [20,21,23]. The biggest disadvantage of this type of radiation source is the fact that it typically cannot maintain a constant microwave power [25]. Using a single-mode microwave reactor allowed starch to undergo the dextrinization process under controlled heating conditions at microwave power up to 300 W [26]. At the same time, it was shown that heating with higher microwave power could cause overheating of the reaction mixture [27]. In order to avoid the risk of overheating, the use of intermittent heating has been proposed [28].

To the best of our knowledge, this article presents, for the first time, a comprehensive and comparative study on dietary fiber obtained from potato starch under continuous and discontinuous microwave heating conditions. It was hypothesized that the discontinuous process would be more effective than the continuous one. In order to confirm this hypothesis, the dextrins obtained by both methods were subjected to structural and physicochemical tests and their properties were compared.

## 2. Results and Discussion

### 2.1. Solubility of Dextrins

**Figure 1:**
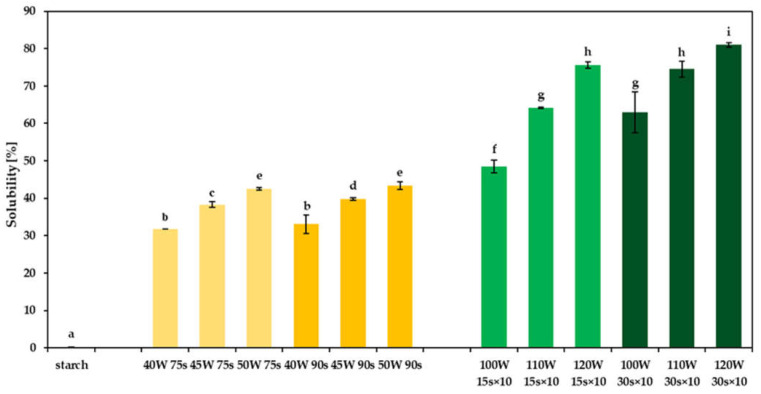
Solubility of dextrins prepared by continuous (yellow) and discontinuous (green) microwave-assisted heating. Different superscript lowercase letters (a, b, …) indicate significant differences (*p* < 0.05) between each dextrin.

The solubility of dextrins at 20 °C was affected by the microwave-assisted heating conditions, including the microwave power intensity and the processing time (Figure 1). When continuous heating was carried out, the lowest solubility (31.8%) was observed for the 40 W 75 s sample, while the 50 W 90 s dextrin was the one with the highest solubility (43.4%). When using discontinuous heating, the lowest solubility (48.5%) was determined for the sample obtained at 100 W for 15 s, and the highest (81.0%) for dextrin prepared at 120 W for 30 s. The dextrins obtained by the discontinuous heating showed higher solubility than dextrins prepared by the continuous heating. Given the same processing time, the higher the radiation power was, the higher the solubility of dextrins was. Moreover, the solubility increased with increasing heating time. Modification of starch caused by microwave-assisted heating in the presence of acids resulted in an increase in solubility, similar to dextrins obtained by using conventional heating in the presence of acids [8,16,29]. This might be due to the hydrolysis reaction during pyroconversion [8,13,30]. The high solubility of dextrins obtained from starch using microwave heating can ensure good homogenization of dextrins in the aqueous environment of food products [31].

### 2.2. Dextrins Dextrose Equivalents (DE)

**Figure 2:**
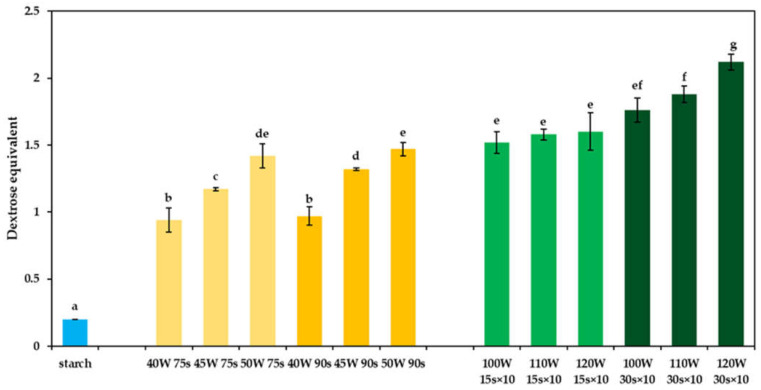
Dextrose equivalent of potato starch (blue) and dextrins prepared by continuous (yellow) and discontinuous (green) microwave-assisted heating. Different superscript lowercase letters (a, b, …) indicate significant differences (*p* < 0.05) between each dextrin.

Although the dextrose equivalents (DE) values (Figure 2) were low for both dextrins obtained by continuous and discontinuous microwave-assisted heating, the ones prepared by the discontinuous heating showed relatively higher DE values (DE = 1.52–2.12) than the dextrins obtained by continuous heating (DE = 0.94–1.47). To have dextrins with such low DE values is important when it is planned to add dextrins into healthy foods without the addition of a high amount of sugars [16]. It can be assumed that, during the heating, a significant depolymerization of starch occurred, as the DE for native potato starch was only 0.2. It is well known that dextrinization processes significantly increase DE values [12]. A higher modification level of dextrins obtained by discontinuous microwave-assisted heating was confirmed also by their higher solubility values, as shown earlier [24].

### 2.3. Color Parameters (L* a* b*)

**Figure 3:**
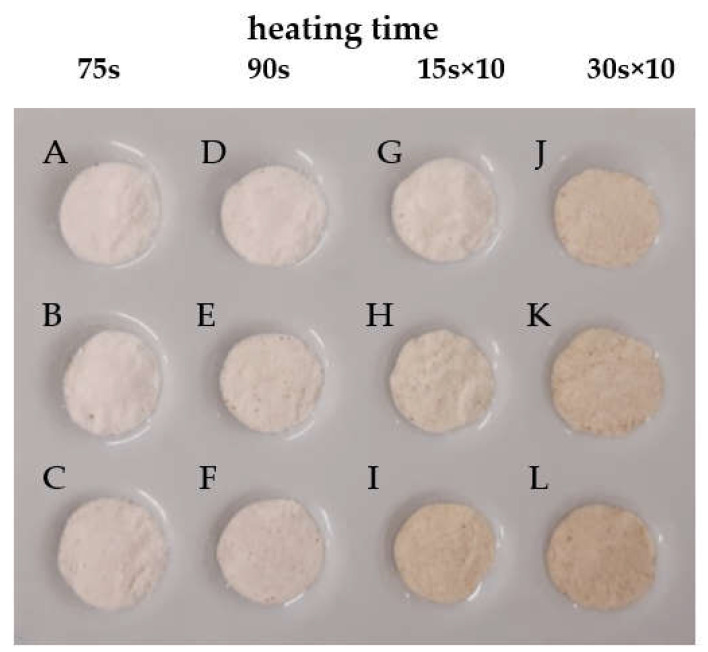
Appearance of dextrins obtained by microwave-assisted heating: continuous—40 W (**A**,**D**), 45 W (**B**,**E**), and 50 W (**C**,**F**) and discontinuous–100 W (**G**,**J**), 110 W (**H**,**K**), and 120 W (**I**,**L**).

After exposure to microwave irradiation, the white color of native potato starch changed (Table 1). All dextrins were characterized by the easily visible color differences, when compared with standard native potato starch, as evidenced by ∆E values higher than 5 [13]. Depending on the processing conditions, dextrins with coloration ranging from cream to brown-yellowish were obtained (Figure 3). Positive parameters a* and b* confirm share of red and yellow colors of dextrins The reduction in whiteness and predominance of beige/brown can be related to the progress of the caramelization reaction [12,32,33]. It can be concluded that increasing both the microwave power intensity and the duration of heating allowed to obtain dextrins with an increasingly darker coloration, which is indicated by even lower values of the L* parameter and higher values of the ∆E parameter. This is in line with previous observations, which showed that harsher conditions (e.g., longer reaction time, higher temperature, or acid concentration) resulted in a darker color of dextrins [8,12,13,34].

### 2.4. Total Dietary Fiber Content

**Figure 4:**
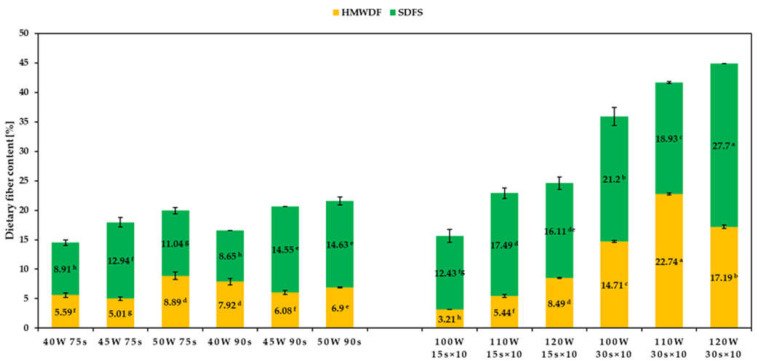
Dietary fiber content of dextrins prepared by continuous (**left**) and discontinuous (**right**) microwave-assisted heating. Different superscript lowercase letters (a, b, …) in the same column indicate significant differences (*p* < 0.05) between each parameter for each dextrin.

Total dietary fiber content for dextrins obtained by continuous microwave-assisted heating ranged from 14.5% to 21.5% (Figure 4), which was unsatisfactorily low. All dextrins obtained by discontinuous heating were characterized by higher DF content, except dextrin 100 W 15 s (TDF = 15.6%) prepared in the mildest conditions among samples heated 10 times. The TDF content increased both with the increasing of microwave power intensity and by extending the heating time. That is in line with other previously published results, where increasing content of non-digestible carbohydrates with increasing process intensity was observed [13,35,36]. The highest TDF content amounted to 45%, determined for the dextrins prepared by discontinuous heating of starch at 120 W for 30 s. This may indicate that the use of microwave-assisted discontinuous heating allows to obtain dextrins with even twice the content of dietary fiber than dextrins obtained by convectional heating of potato starch acidified with the same amounts of hydrochloric and citric acids [11]. Additionally, the proportion in dextrins of high-molecular weight dietary fiber (HMWDF) and low molecular weight dietary fiber soluble in water and not precipitated in 78% aqueous ethanol (SDFS) varied depending on the conditions of the heating process. In almost all obtained dextrins, the fraction of compounds of low molecular weight constituted the majority. Similar results can be seen in the case of studies conducted by other authors [35,36].

### 2.5. SEM

**Figure 5:**
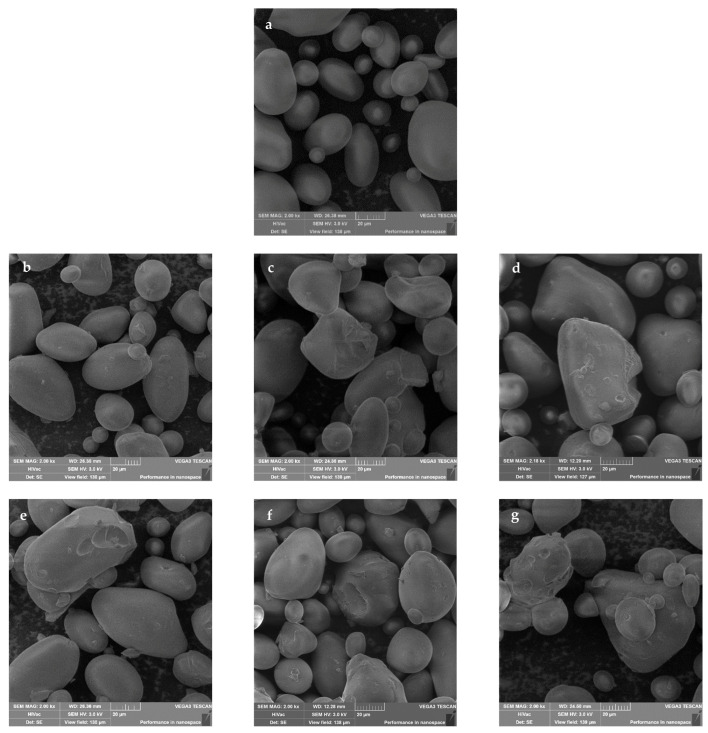
SEM micrographs of native potato starch (**a**) and obtained dextrins obtained by microwave-assisted heating for 15 s at 100 W (**b**), 110 W (**c**), and 120 W (**d**) and for 30 s at 100 W (**e**), 110 W (**f**), and 120 W (**g**).

Figure 5 shows the influence of microwave-assisted heating on the shape of potato starch granules. In the native form, the potato starch was a mixture of smooth spherical shape granules with a size lower than 20 µm and oval shape granules with sizes ranging from 30 µm to 60 µm, possessing edges clearly visible and without damage on their surfaces (Figure 5a). All these characteristics are specific to granules of native potato starch [37]. For starch heated once for 75 s, regardless the microwave power intensity used, the starch granular integrity was maintained. Additionally, single microwave-assisted heating for 90 s did not cause any significant change in the granular morphology. When exposed to the discontinuous heating, the granular shape of starch was altered (Figure 5b–g). Noticeable damage on the surface of the granules was observed, while preserving their granular nature, size, and shape. The greatest damage was observed on the surface of larger starch granules. This might be due to the fact that it is easier to gelatinize the larger starch granules than the smaller ones that have higher gelatinization temperatures, thus requiring higher energy and/or processing time till the granules achieve swelling and rupture [38]. Moreover, the magnitude of observed changes increased with the extension of the heating time and the increase in the microwave power intensity. For dextrin obtained by heating 10 times at 120 W for 30 s, aggregation of starch granules into lumps was observed. This is consistent with the results of other authors concerning changes in starch granules during dry heating treatment [39].

### 2.6. X-ray Diffraction (XRD)

**Figure 6:**
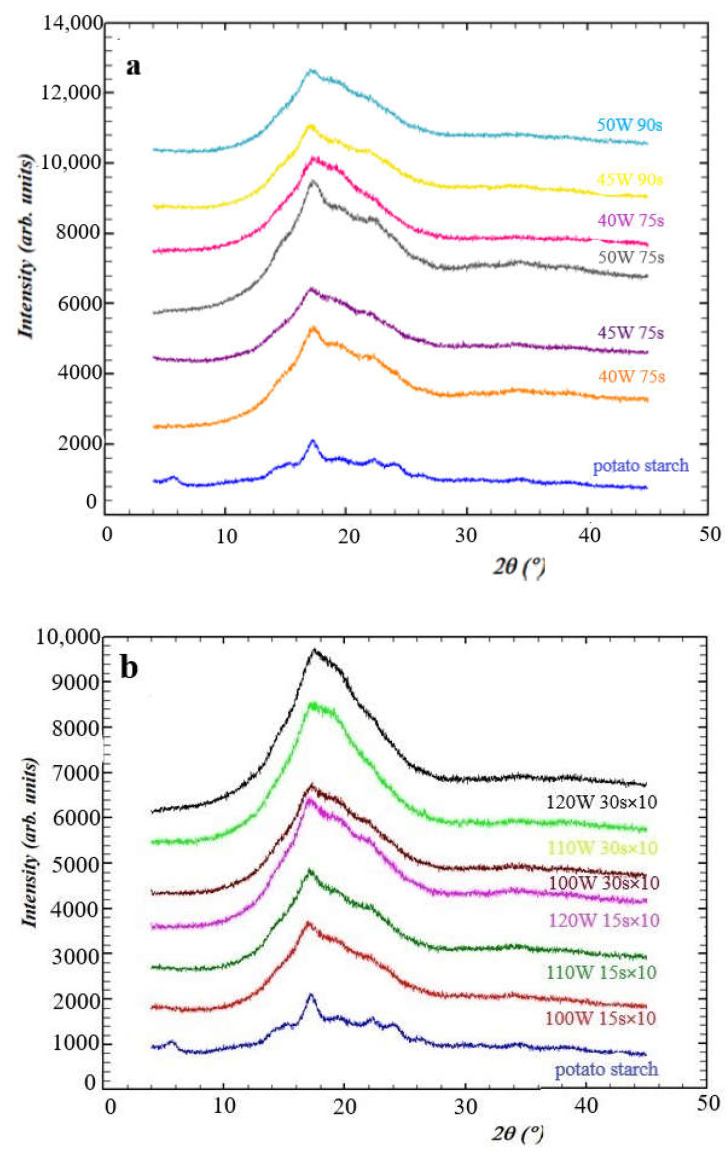
X-ray diffraction patterns of dextrins obtained after heating potato starch under continuous (**a**) and discontinuous (**b**) microwave irradiation.

The XRD pattern for potato starch contained diffraction peaks (2θ) at 5.59°, 15.12°, 17.21°, 19.54°, 22.40°, 23.90°, and 26.27° (Figure 6), which correspond to the B-type crystalline structure of potato starch [40,41,42]. The potato starch used in this study showed a crystallinity index (Xc) of 0.47. Regardless of the type of microwave-assisted heating used (continuous or discontinuous), a decrease in crystallinity for all the obtained dextrins was observed (Table 2).

For potato starch continuously heated (Figure 6a), a 2-fold (even 3 times for one sample) decrease in crystallinity was observed, compared with the native potato starch. For potato starch heated once for 75 s in the microwave reactor, the degree of crystallinity decreased with microwave power increasing. The same behavior was observed for dextrins obtained after heating for 90 s. Comparing the compounds subjected to the same power and different heating times, a slight decrease in the crystallinity of dextrins obtained in the longer heating was observed. For discontinuous heating conditions (Figure 6b), the crystallinity index decreased approximately twice for dextrins obtained at 100 W and 110 W for 15 s and much more for subsequent dextrins—up to almost 7 times for the last one obtained at 120 W for 30 s. For dextrins heated 10 times for 15 s or 30 s, it was observed that the higher the power, the lower the crystallinity degree. The crystallinity degree was also influenced by the heating time at a given power. For dextrins heated for 30 s, the crystalline form share was approximately twice as small as in dextrins heated for 15 s. The degree of crystallinity for dextrins heated 10 times for shorter periods of time was lower than for dextrins heated once for longer time. The presented results are in line with the observations of other authors who showed unambiguously that modification of native potato starch by microwave heating at a certain power and time decreased the crystallinity index, which was observed as a decrease or absolute disappearance of diffraction reflections typical for B-type X-ray diffraction pattern [43,44]. Consequently, this led to an increase in the amorphous form. The percentage crystallinity decrease verified during dextrinization was also similar to that observed by other authors [30,45,46,47].

### 2.7. Thermal Properties of Dextrins (DSC)

For dextrins obtained under all tested operational conditions, the temperature values of T_o_, T_p_, and T_c_ were significantly higher than the ones obtained for the native potato starch (Table 3). These thermal changes indicate that the applied microwave-assisted heating parameters significantly affected the potato starch structure reorganization. T_o_, T_p_, and T_c_ temperatures characterize the susceptibility of starch to gelatinize and are known to depend on the intra-granular interactions strength [48]. High T_o_ and T_p_ values mean that more energy is required to initiate the starch gelatinization [49]. The obtained results suggest that dextrinization process caused significant changes in the starch crystalline region, resulting in a narrower endothermic gelatinization peak (lower ΔT values compared with the native starch) with a lower enthalpy value. Dextrinization, reducing the crystallinity of dextrins (Table 2), simultaneously increased their solubility (Figure 1), dextrose equivalent value (Figure 2), and the share of SDFS soluble dietary fiber (Figure 4). It can be assumed that during the modification of starch, less perfect crystallites with short double helices were formed [50]. A good example of clear changes in the crystal structure of starch during its modification are the samples dextrinized under the following conditions: 120 W 15 s × 10 as well as 110 W and 120 W 30 s × 10, which had the highest dissolving power in water (Figure 1) among all tested dextrins. For these trials, no endothermic transformation during heating was observed in the DSC analysis, indicating the loss of starch crystallinity and gelatinization ability. All depolymerization products obtained showed significantly lower values of ΔH than those for native potato starch. The values of ΔH are correlated with the degree of starch crystallinity because melting of crystallites (formed by amylopectin) requires more energy [48]. As we have shown, stronger processing conditions, including high microwave power intensity and long operating times, and the method of discontinuous heating, can cause a greater degree of hydrolysis, resulting in a complete loss of the ordered structure of the starch. The greater degree of starch depolymerization resulted, first of all, in a significant increase in the soluble fiber in the tested dextrins and in an increase in their solubility compared with native starch. Based on the thermal analysis data of dextrins, when compared with native potato starch, there was a clear influence of the applied microwave heating power, the processing time, and method of microwave operation (continuous or discontinuous heating) on the degradation of potato starch structure.

### 2.8. Glycosidic-Linkage Analysis

According to GC-MS analysis, native potato starch contained more than 90% of (1 → 4)-linked Glc*p* and small amounts of terminal and (1 → 4,6)-linked Glc*p* (Table 4). For all dextrin samples, a significant decrease in the percentage of (1 → 4)-Glc*p* and a marked increase in terminal and (1 → 6)-Glc*p* were observed. The results are in line with observations of Nunes et al. [51], who used dry thermal treatments at 265 °C for amylose and amylopectin. The majority of the samples contained small amounts of (1 → 2) and (1 → 3)-Glc*p*. In the context of dextrins’ resistance to enzymatic digestion, in addition to the presence of such bonds, the presence of branched molecules, i.e., with more than two groups -OH involved in the formation of glycosidic bonds, is also beneficial. In each sample low amounts of (1 → 2,4) and (1 → 3,4)-Glc*p* were presented, and in some dextrin samples it was also possibly to quantify small amounts of (1 → 2,6)-Glc*p*. Increasing microwave power intensity and heating time seemed to favor the molecules branching. For dextrins prepared under continuous and discontinuous microwave-assisted heating, the presence of molecules other than (1 → 4)-Glc*p*, terminal, and (1 → 4,6)-Glc*p* differed depending on the modification conditions used. The values of the relative percentage of non-starch glycosidic linkages ranged from 4.6% to 7.2% for samples heated once for 75 s; 8.0% to 9.2% for samples heated once for 90 s; 10.6% to 12.3% for samples heated 10 times for 15 s; and 13.4% to 17.3% for samples heated 10 times for 30 s. These results were in line with the 17.8% and 5.8% of linkages other than (α1 → 4) reported by Bai and Shi [52] for pyrodextrin and maltodextrin samples obtained from waxy maize starch.

## 3. Materials and Methods

### 3.1. Materials

Potato starch and analytical grade reagents were purchased from Sigma-Aldrich, Poznan, Poland; enzymatic kits were purchased from Megazyme, Wicklow, Ireland.

### 3.2. Preparation of Dextrins (Continuous and Discountinuous Process) Using Microwave-Assisted Heating

Dextrins were prepared by weighing 80 g of potato starch, spreading it onto a glass tray, and spraying it with 0.5% (*v*/*v*) solutions of hydrochloric and citric acids to a final concentration of both acids amounting to 0.1% *w*/*w* related to dry starch basis. Acidified starch was mixed and distributed on the surface of the tray. The prepared material was dried at 110 °C for 2 h in order to obtain a final water content of less than 5%. Afterwards, it was weighed (5 g) into 35 mL glass vessels and heated in a Discover SP microwave reactor (CEM Corporation, Matthews, NC, USA) at 40 W, 45 W or 50 W for 75 s or 90 s, for the continuous process, and at 100 W, 110 W or 120 W for 15 s or 30 s, for the discontinuous process. During the continuous process, the samples inside the vessels were heated once, while during the discontinuous process each of the samples inside the vessels was heated 10 times under the selected conditions (the vessels’ content was mixed after each heating cycle to increase uniformity of microwave heating and modification of starch level). The different microwave irradiation conditions used in the study and operational advantages and disadvantages are presented in Table 5. Conditions were proposed based on screening tests conducted on a large group of samples. These studies clearly showed that the mildest conditions resulted in white, non-dextrinized samples, while the most extreme conditions favored a caramelization process. Additionally, from our preliminary studies, we found a correlation between lightness and total dietary fiber content (McPearson coefficient was −0.872). For this reason, samples could be preliminary selected by color screening. Finally, six dextrins using continuous microwave heating and six by discontinuous heating were prepared and subjected to further analyses.

### 3.3. Solubility of Dextrins

Solubility in water at 20 °C was measured according to Richter’s method [53]. Dextrins in the amount 0.5 g were suspended in 40 mL of distilled water and were stirred at 20 °C for 30 min. The suspension was subsequently centrifuged at 21,381× *g* for 10 min, and 10 mL of supernatant was transferred into weighing vessels of known weight. Then, vessels with supernatants were dried to constant weight at 130 °C. Afterwards, the obtained residue was weighed and the solubility (S) in water was calculated using Equation (1):

S = (100 × 40 × b)/(10 × a) [%],(1)

where: a—sample weight, b—weight of residue after drying, volume of evaporated supernatant (10 mL), and total volume of added water (40 mL).

### 3.4. Dextrose Equivalent (DE) of Dextrins

Dextrose equivalent of dextrins was determined using Schoorl–Regenbogen’s method [54]. Dextrins were weighed (0.5 g), suspended in distilled water (10 mL), and then stirred at room temperature for 30 min. Then, Fehling’s solution I (10 mL), Fehling’s solution II (10 mL), and distilled water (20 mL) were added, and the mixtures were brought to a boil in 3 min and boiled for 2 min. After cooling down, potassium iodide (10 mL), sulfuric acid (10 mL), and starch colloidal solution (5 mL) were added, and the mixtures were titrated with sodium thiosulfate. The blank tests were carried out analogically with distilled water. Dextrose equivalent of dextrins was calculated according to [54].

### 3.5. Color Parameters (L* a* b*)

The color of dextrins was measured using a Chroma Meter CR-400 (Konica Minolta Sensing, Osaka, Japan). L* (luminosity), a* (red/green color), b* (yellow/blue color) components were determined with the CIELab color profile. Color difference was calculated from Equation (2):

(2) $$\Delta E=\sqrt{{\left(\Delta L^{*}\right)}^{2}+{\left(\Delta a^{*}\right)}^{2}+{\left(\Delta b^{*}\right)}^{2}}$$

where: $\Delta L^{*}$, $\Delta a^{*}$, $\Delta b^{*}$ were the differences in the values of L*, a*, b* between native starch and dextrins, respectively. Measurements were performed 10 times for each sample.

### 3.6. Total Dietary Fiber Content According to AOAC 2009.01 Method

High molecular weight dietary fiber (HMWDF), comprising insoluble dietary fiber (IDF) and dietary fiber soluble in water but precipitated in 78% aqueous ethanol (SDFP), and dietary fiber soluble in water and not precipitated in 78% aqueous ethanol (SDFS) were determined following the AOAC 2009.01 method [55]. Briefly, the samples were suspended in ethanol and digested with pancreatic α-amylase/amyloglucosidase mixture in maleic buffer (50 mM) for 16 h at 37 °C. The enzymes were inactivated by using TRIS buffer (0.75 M) and boiling. In the next step, the proteins were digested with protease for 30 min at 60 °C. The enzymes were inactivated by using acetic acid (2 M). Then, ethanol was added to form the HMWDF precipitate. After 1 h, the samples were filtered through vacuum to constant weight and weighed crucibles with diatomaceous earth. The filtrate was recovered for SDFS determination. Residues of HMWDF were washed and dried overnight and then used for determination of proteins and ash content. The recovered filtrate was concentrated, deionized, and analyzed with HPLC. Obtained results were used to determine HMWDF and SDFS content in dextrins.

### 3.7. SEM

The granular shape and surface morphology of native potato starch and the prepared dextrins were observed using a Tescan VEGA 3SBU scanning electron microscope (Tescan, Brno, Czech Republic). The accelerating voltage was 3 kV. Adhesive tape was attached to circular stubs, then all samples were sprinkled onto tape without coating with any conductive material. All samples were observed, and micrographs were taken at magnification of ×2000.

### 3.8. X-ray Diffraction (XRD)

**Figure 7:**
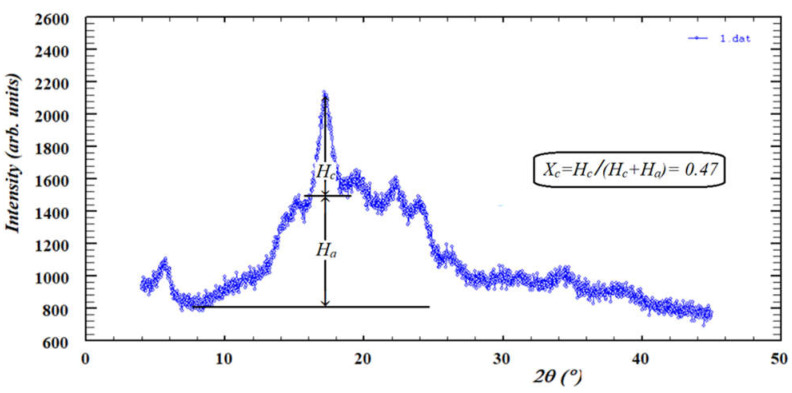
X-ray diffraction patterns for native potato starch. *H_a_* and *H_c_* are the amorphous and crystalline profile heights, respectively.

Phase analysis of dextrins was carried out using powder X-ray diffraction (XRD) Rigaku Miniflex D 600 diffractometer (Rigaku, Tokyo, Japan) with D/teX Ultra silicon strip detector, Cu-Kα radiation. To assess the crystallinity, the method described by Hulleman et al. [56] was used. The values of the crystallinity index (*X_c_*) for all samples were obtained using Equation (3):

(3) $$X_{c}=\frac{H_{c}}{\left(H_{c}+H_{a}\right)}$$

where *H_c_* and *H_a_* are the intensities for the crystalline and amorphous profiles with typical diffraction reflex (121) at a value of 2*θ* between 17° and 18°, as shown in Figure 7, respectively. XRD of native potato starch was used as control.

### 3.9. Thermal Properties of Dextrins (DSC)

Gelatinization properties of native starch and dextrins were determined by differential scanning calorimetry (DSC), following a previously described method, but with some modifications [16,57]. For this purpose, a MICRO DSC III differential scanning calorimeter from Setaram Instrumentation (Caluire, France) was used. Triplicate starch samples (approximately 40 mg) were weighed in stainless steel, high-pressure type ‘batch’ cell at the water/starch ratio of 70:30 (*w*/*w*). Samples were heated from 10 °C to 120 °C at 3 °C min^−1^. The onset (T_o_), peak (T_p_), and conclusion (T_c_) temperatures; gelatinization temperaturę range ΔT_r_ = (T_c_ − T_o_); and enthalpy change (ΔH) expressed in J g^−1^ dry starch were calculated from thermograms.

### 3.10. Methylation Analysis

For determination of glycosidic linkages composition, dextrins were converted to partially O-methylated alditol acetylates [51]. Briefly, dextrins were dissolved in DMSO overnight, then pellets of NaOH were added, and the solutions were mixed for 30 min. Then 80 μL of CH_3_I was added and allowed to react in room temperature under vigorous stirring. After 20 min, 2 mL of distilled water was added, and the solutions were neutralized with 1 M HCl. Subsequently 3 mL of dichloromethane was added, and the solutions were manually shaken and further centrifuged at 11,600× *g*. The water phase was removed, and the precipitate was washed twice with distilled water. The samples were evaporated to dryness. To ensure a complete methylation, this step was repeated. Afterwards, the samples were hydrolyzed with trifluoroacetic acid (TFA) at 121 °C for 1 h, with subsequent evaporation of the acid. For the carbonyl reduction, 300 μL of 2 M NH_3_ and 20 mg of sodium borodeuteride were added. The mixtures were incubated at 30 °C for 1 h and then excess of borodeuteride was removed by addition of glacial acetic acid. The partially methylated alditols were acetylated by adding 1-methylimidazole and acetic anhydride. After 30 min at 30 °C, the excess of acetic anhydride was removed and the partially O-methylated alditol acetates (PMAA) were extracted with dichloromethane in two steps. Then, the dichloromethane was evaporated, and the samples were dissolved in anhydrous acetone, which was evaporated prior the GC-MS analysis.

### 3.11. GC-MS Analysis

PMAA obtained in the Section 3.9 were analyzed by gas chromatography mass spectrometry (GC–MS) using an Agilent Technologies 6890N Network (Santa Clara, CA, USA). The GC was equipped with a DB-1 column (J&W Scientific, Folsom, CA, USA). 0.2 μL of the samples were injected with the injector operating at 220 °C. The helium carrier gas had a flow rate of 0.2 mL/min. The GC was connected to an Agilent 5973 (Santa Clara, CA, USA) mass quadrupole selective detector.

### 3.12. Statistical Analysis

The results were subjected to statistical analysis using Statistica 13.3 software (StatSoft, Tulsa, OK, USA). A completely randomized design was applied for all the experiments. Analysis of variance was performed. Mean comparison was done using Duncan’s new multiple range test. All assays, except for color measurement (where the tests were repeated 10 times), were performed in triplicate, and their results were averaged if the difference was not statistically significant at *p* < 0.05.

## 4. Conclusions

Dextrinization of starch acidified with hydrochloric and citric acids using microwave radiation in a single-mode reactor was successfully carried out. The use of a single-mode microwave reactor allowed high repeatability of conducted processes to be created. The use of discontinuous process (10-fold heating with mixing between cycles) proved to be more effective than the continuous one (single heating). The dextrins obtained by discontinuous heating showed higher solubility and higher content of, among others, (1 ⟶ 2), (1 ⟶ 3), (1 ⟶ 4,6), (1 ⟶ 2,4), and (1 ⟶ 3,4) Glc*p* linkages, absent in the native starch, thus highlighting the higher total dietary fiber content. Moreover, the discontinuous heating decreased the starch crystallinity, changing the granules surface morphology and originating samples with higher dextrose equivalent values and darkest coloration, thus revealing a more pronounced degree of starch modification. The applied dextrinization processes allowed products with a high total dietary fiber content to be obtained.

## Figures and Tables

**Table 1:** Color parameters of dextrins obtained after exposure of potato starch to continuous and discontinuous microwave-assisted heating, under acidic conditions.

Dextrin	L*	a*	b*	Δ*E*
Native potato starch	94.06	0.07	1.82	
40 W 75 s	88.89 ± 0.14 ^a^	0.56 ± 0.03 ^j^	9.89 ± 0.11 ^h^	9.59 ± 0.10 ^i^
45 W 75 s	87.31 ± 0.13 ^c^	1.00 ± 0.04 ^g^	10.28 ± 0.12 ^g^	10.86 ± 0.07 ^h^
50 W 75 s	86.69 ± 0.12 ^d^	1.37 ± 0.04 ^f^	11.14 ± 0.11 ^d^	11.95 ± 0.10 ^f^
40 W 90 s	88.00 ± 0.12 ^b^	0.99 ± 0.04 ^g^	10.91 ± 0.08 ^e^	10.96 ± 0.08 ^h^
45 W 90 s	86.33 ± 0.09 ^d^	1.49 ± 0.03 ^e^	10.91 ± 0.07 ^e^	12.02 ± 0.07 ^f^
50 W 90 s	85.61 ± 0.14 ^e^	1.52 ± 0.02 ^e^	10.67 ± 0.12 ^f^	12.32 ± 0.06 ^e^
100 W 15 s × 10	88.21 ± 0.09 ^b^	0.67 ± 0.07 ^h^	8.56 ± 0.11 ^i^	8.69 ± 0.08 ^j^
110 W 15 s × 10	86.95 ± 0.23 ^d^	0.6 ± 0.05 ^i^	10.94 ± 0.26 ^def^	11.41 ± 0.23 ^g^
120 W 15 s × 10	80.44 ± 0.20 ^g^	2.13 ± 0.05 ^d^	12.87 ± 0.15 ^c^	17.26 ± 0.12 ^d^
100 W 30 s × 10	81.12 ± 0.18 ^f^	2.32 ± 0.08 ^c^	14.64 ± 0.16 ^b^	18.03 ± 0.19 ^c^
110 W 30 s × 10	79.78 ± 0.11 ^h^	2.71 ± 0.06 ^b^	15.29 ± 0.22 ^a^	19.47 ± 0.10 ^b^
120 W 30 s × 10	79.38 ± 0.25 ^i^	3.07 ± 0.16 ^a^	15.16 ± 0.17 ^a^	19.71 ± 0.12 ^a^

Different superscript lowercase letters (a, b, …) in the same column indicate significant differences (*p* < 0.05) between each parameter for each dextrin.

**Table 2:** Values of crystallinity index (X_c_) dextrins prepared under continuous and discontinuous microwave-assisted heating and native of potato starch.

Sample	X_c_	
Native potato starch	0.47	
40 W 75 s	0.28	Continuous heating
45 W 75 s	0.23	
50 W 75 s	0.21	
40 W 90 s	0.24	
45 W 90 s	0.18	
50 W 90 s	0.17	
100 W 15 s × 10	0.24	
110 W 15 s × 10	0.22	Discontinuous heating
120 W 15 s × 10	0.14	
100 W 30 s × 10	0.12	
110 W 30 s × 10	0.10	
120 W 30 s × 10	0.07	

**Table 3:** Gelatinization parameters of dextrins prepared under continuous and discontinuous microwave-assisted heating and native of potato starch.

Dextrin	To	Tp	Tc	ΔTr	ΔH
	(°C)				(J g^−1^ d.w.)
Native starch	58.76 ± 0.14 ^a^	63.23 ± 0.11 ^a^	72.33 ± 0.15 ^a^	13.57 ± 0.11 ^a^	14.30 ± 0.25 ^a^
40 W 75 s	60.19 ± 0.22 ^bAA^*	64.95 ± 0.16 ^bAA^*	73.01 ± 0.10 ^bAA^*	12.82 ± 0.17 ^bAA^*	8.37 ± 0.17 ^bAA^*
45 W 75 s	62.59 ± 0.21 ^cBA^*	66.80 ± 0.20 ^cBA^*	74.50 ± 0.14 ^cBA^*	11.91 ± 0.10 ^cBA^*	7.90 ± 0.19 ^cBA^*
50 W 75 s	63.34 ± 0.17 ^dCA^*	67.21 ± 0.19 ^dCA^*	74.96 ± 0.21 ^dCA^*	11.62 ± 0.18 ^dCA^*	4.48 ± 0.15 ^dCA^*
40 W 90 s	62.35 ± 0.20 ^eAB^*	70.10 ± 0.12 ^eAB^*	75.03 ± 0.11 ^eAB^*	11.22 ± 0.14 ^eAB^*	3.09 ± 0.11 ^eAB^*
45 W 90 s	65.87 ± 0.15 ^fBB^*	71.88 ± 0.21 ^fBB^*	75.97 ± 0.27 ^fBB^*	10.10 ± 0.17 ^fBB^*	1.50 ± 0.21 ^fBB^*
50 W 90 s	68.96 ± 0.14 ^gCB^*	73.90 ± 0.27 ^gCB^*	76.91 ± 0.21 ^gCB^*	7.95 ± 0.20 ^gCB^*	0.50 ± 0.19 ^gCB^*
100 W 15 s × 10	59.17 ± 0.21 ^hAA^*	73.85 ± 0.54 ^hAA^*	76,74 ± 0.23 ^hAA^*	17.57 ± 0.21 ^hAA^*	1.30 ± 0.17 ^hAA^*
110 W 15 s × 10	60.75 ± 0.33 ^iBA^*	66.96 ± 0.16 ^iBA^*	69.55 ± 0.21 ^iBA^*	8.80 ± 0.17 ^iBA^*	0.33 ± 0.15 ^iBA^*
120 W 15 s × 10			No endothermic peak		
100 W 30 s × 10	68.23 ± 0.12 ^jAB^*	70.37 ± 0.24 ^jAB^*	72.97 ± 0.21 ^jAB^*	4.74 ± 0.24 ^jAB^*	0.43 ± 0.10 ^jAB^*
110 W 30 s × 10			No endothermic peak		
120 W 30 s × 10			No endothermic peak		

To, onset temperature; Tp, peak temperature; Tc, conclusion temperature; ∆Tr, gelatinization temperature range = (Tc − To); ∆H, enthalpy expressed in J g^−1^ dry starch. Different superscript lowercase letters (a, b, …) in the same column indicate significant differences (*p* < 0.05) between each parameter for each dextrin compared with native starch. Different superscript uppercase letters (A, B or C) in the same column indicate significant differences (*p* < 0.05) between each parameter for dextrins from a given series, depending on the microwave power and operating time. Different superscript uppercase letters with asterisk (A* or B*) in the same column indicate significant differences (*p* < 0.05) between each parameter for dextrins with the same microwave power but different operating times.

**Table 4:** A percentage of different glycosidic linkages of dextrins prepared under continuous and discontinuous microwave-assisted heating and native of potato starch.

Sample	t-Glc*p*	2-Glc*p*	3-Glc*p*	4-Glc*p*	6-Glc*p*	2,3-Glc*p*	2,4-Glc*p*	2,6-Glc*p*	3,4-Glc*p*	4,6-Glc*p*	2,3,6-Glc*p*	2,4,6-Glc*p*	3,4,6-Glc*p*
potato starch	3.9			93.0						3.1			
40 W 75 s	9.2			85.6	0.6		0.3		0.3	4.1			
45 W 75 s	10.0			84.9	0.5		0.3		0.2	4.2			
50 W 75 s	11.0	0.4	0.4	79.0	2.3		0.6		0.4	5.9			
40 W 90 s	12.9	0.6	0.4	77.4	1.6		0.5	0.2	0.5	5.6		0.1	0.2
45 W 90 s	10.6	0.6	0.3	79.7	1.5		0.6	0.1	0.5	5.9		0.1	0.1
50 W 90 s	8.2	0.6	0.2	81.5	1.2		0.4	3.3	0.6	3.5	0.1	0.2	0.2
100 W 15 s × 10	12.4	2.4	1.4	75.8	1.2		0.5	0.1	0.4	5.8			
110 W 15 s × 10	15.3	0.7	0.7	68.8	4.4		0.8		0.6	8.7			
120 W 15 s × 10	17.4	0.8	0.8	65.0	5.3		0.9		0.6	9.1			
100 W 30 s × 10	16.2	1.0	0.8	65.0	5.3		0.9		0.6	9.8		0.2	0.2
110 W 30 s × 10	17.9	1.2	1.1	59.6	7.1		1.2		0.8	11.0			0.3
120 W 30 s × 10	16.4	1.8	1.1	62.4	3.1	0.1	1.4	0.3	1.0	11.3	0.1	0.5	0.5

**Table 5:** Microwave irradiation conditions.

	Continuous Heating	Discontinuous Heating
Processing conditions	40 W, 45 W, or 50 W for 75 or 90 s (1 time processed)	100 W, 110 W or 120 W for 15 s or 30 s (10 times processed)
Advantages	simple, fast, one-step process, better repeatability	high degree of possible modification, more homogeneous samples
Disadvantages	more heterogeneous samples, low degree of possible modification	longer, 10-step process, worse repeatability

## Data Availability

All data are included in the article.

## Sample Availability

Not available.
